# Endodontic Emergencies Encountered in a Tertiary Dental Hospital during the Periods of Prelockdown, Lockdown, and Sequential Unlock Phases of the First Wave of COVID-19 in Ahmedabad City, India

**DOI:** 10.1155/2022/5856267

**Published:** 2022-02-08

**Authors:** Akshayraj Langaliya, Dolly Patel, Aarshvi Shah, Aravind Kumbhar, Jinali Shah, Sharan Shah, Ishan Bhatt, Dhruvisha Dayala, Rajmeet Singh Gill, Saloni Patel, Yatra Patel, Mandar Shah, Mihika Shah, Rushvi Patel, Aashvi Agarwal, Drashti Panchal, Vishwani Vachhani

**Affiliations:** ^1^Department of Conservative Dentistry and Endodontics, AMC Dental College and Hospital, Bhalakhiya Mill Compound, Opp. Anupam Cinema, Ahmedabad 380008, India; ^2^AMC Dental College and Hospital, Bhalakhiya Mill Compound, Opp. Anupam Cinema, Ahmedabad 380008, India; ^3^Smt. N H L Municipal Medical College, V S Hospital Compound, Ashram Road, Ahmedabad 380 006, India

## Abstract

**Introduction:**

With the worldwide spread of SARS-CoV-2 infection, temporary suspension of all the elective dental procedures and an urgent shift to nonaerosol producing dental treatments were observed. This study provides a detailed description of emergency treatments provided in the Department of Endodontics at a tertiary healthcare centre during the period of prelockdown, lockdown, and sequential unlocking from March 1, 2020, to October 31, 2020.

**Methods:**

Access to General and Departmental OPD data along with treatment records was obtained and was segregated based on age, sex, and treatments performed. Treatments were divided into aerosol generating procedures (AGPs) and non-AGPs and further subdivided into palliative treatment (PT), pulp capping (PC), incision and drainage (ID), temporary filling (TF), pulpectomy (PU), and pulpotomy (PO). Data was analysed and subjected to chi-square test.

**Results:**

A total of 15052 patients approached general OPD during the period of 8 months of which 5698 (37.86%) were endodontic in origin and treatments offered were PT 858 (15.05%), PO 1560 (27.37%), PU 2018 (35.42%), TF 500 (8.78%), ID 164 (2.88%), and PC 598 (10.94%). Also, more females (57.28% (3264/5698)) visited the department as compared to males (42.72% (2434/5698)).

**Conclusion:**

The pandemic had turned the tables on over the people around the world, and it has become extremely necessary to rule out the emergencies needed to treat the patients accordingly shifting more towards non-AGPs compared to AGPs among the various age groups of the society.

## 1. Introduction

Coronavirus is a single-stranded RNA virus that belongs to Coronaviridae family [1] of *Betacoronavirus* type infecting mainly gastrointestinal tract, respiratory tract, and central nervous system in humans and mammals [2]and has an incubation period of 1–14 days with mild to severe symptoms like sore throat, cough, shortness of breath, loss of taste and smell, even multiple organ failure, and septic shock, which may even lead to death [3]. It was thought that coronavirus began from the sea food market in China, which began as an epidemic in Wuhan, China, and later on was declared as a pandemic by WHO on March 11, 2020. India, the second most populous country in the world, too could not escape the rapid spread following which a nationwide lockdown was imposed in India on March 21, 2020. CDC's guidelines of Shelter in Peace suggested identification of the disease, isolation, and providing proper treatment to the needy. There was virtually no profession which was not affected by the COVID-19 crisis, including dentistry, particularly due to the fact that dental professionals work in close approximation to oral cavity, a primary source of transmission of COVID-19. Therefore, it became absolutely necessary to curb down all the nonessential services and to provide only the emergency dental treatment with restriction on the use of aerosol generating treatments as it is more prone to transmit the infection [4, 5]. Indian Endodontic Society (IES) released a statement that all the nonemergency elective dental procedures should be postponed keeping only the emergency procedures operational. Recommendations were made to triage patients and to decide about the emergency required minimizing the exposure with proper infection control maintained [6]. Further, American Dental Association (ADA) released clear-cut guidelines defining procedures that should be included as urgent and emergency dental procedures [7]. According to CDC, the treatments provided are divided basically into 2 types, that is, aerosol generating procedures (AGPs) and nonaerosol generating procedures (non-AGPs). During the period of lockdown, it was advised to stop using the AGPs as they could spread the virus demanding a shift towards the non-AGPs dental procedures [8].

Ahmedabad city, governed by the local governing body called AMC, is the fifth most densely populated city of India, which is also considered as financial and industrial hub soon turned into a hotspot booming with cases every single day. AMC launched a campaign “Chase the Virus,” wherein majority of the medical, dental, and paramedical professionals and students of the institutions affiliated to AMC were urgently deployed to work as frontline warriors for treating the patients infected with COVID-19 and to perform mass screening to restrict the spread of infection. AMC Medical Education Trust (AMC-MET) has 4 medical institutions and 1 tertiary dental institution; it was imperative that it being the only dental institution it had to be kept constantly open to provide treatments to the patients as per the need without a single day of shutdown. Even though majority of interns, postgraduates, and staff members of the institute were posted for various COVID-19 duties, the rest were working continuously in the hospital round the clock following the guidelines [7]. The newly adopted guidelines soon became the new normal and continued with the phases of sequential unlock in the coming 5 months and gradually all the dental treatments rebounded to its original fledge with maximum precautions taken. [9].

As per the authors' knowledge, a handful of studies evaluating the dental emergencies in tertiary dental healthcare institution have been published so far; however, this is probably the first of its kind with such a detailed description of all the variables followed up over such a long duration. This study describes the utilization of Ahmedabad Municipal Corporation (AMC) Dental Hospital, which acts as a tertiary dental healthcare hospital during the pandemic highlighting the endodontic emergencies encountered at that time of lockdown and after lockdown.

The primary objectives of this study are as follows:

1. To evaluate the number of patients who availed the emergency dental service during immediate prelockdown, lockdown, and postlockdown period according to the age and gender
2. To understand the distribution of various treatment modalities offered by the tertiary healthcare centre

## 2. Materials and Methodology

The ethical clearance for this retrospective study was not taken as the study is considered as a clinical audit data and no personal information or data of patient was directly received and the treatment performed was all evidence-based.

However, prior permissions from the dean of the institution and head of the administrative department were taken to access the data of general OPD and the patient data as well as treatment records of Department of Conservative Dentistry and Endodontics.

Only the new cases that approached the department were included in the study. Old cases that were approached on follow-up basis or appeared as a result of failure of previous treatment were not considered for this study.

The obtained departmental data was tabulated and classified month-wise on the basis of age, sex, and the type of treatment delivered on each day in Microsoft Excel 2019 over the span of March 2020 to October 2020. The periods were designated as prelockdown from March 1, 2020, to March 20, 2020, lockdown from March 21, 2020, to May 31, 2020, and postlockdown with Unlock 1 from June 1, 2020, to June 30, 2020, Unlock 2 from July 1, 2020, to July 31, 2020, Unlock 3 from August 1, 2020, to August 31, 2020, Unlock 4 from September 1, 2020, to September 30, 2020, and Unlock 5 from October 1, 2020, to October 31, 2020. The collected data was later subjected to statistical analyses for further comparisons.

For this study, AGP is defined as procedure that uses slow or high speed handpiece, ultrasonic scalers, air-water syringes, manipulation of gingiva, and rotary instruments intraorally including extractions, whereas non-AGP is defined as recementation of temporary definitive crowns and bridge without the use of handpiece ART, chemicomechanical caries removal with SDA, IRM, carisolv, caridex and papain gel, and even removal of soft dentinal caries with spoon excavators and restoring the tooth using adhesive materials like GIC, fluoride varnish, and desensitizing agents. Dental emergency was evaluated according to the ADA classification of dental emergency as urgent dental care and non-urgent dental care along with treatment protocol describing about AGP or non-AGP treatment plan [8, 10].

After a thorough screening of each patient, a diagnosis was made, and treatment plan was fabricated on basis of clinical judgement provided by available faculty members based on which palliative approach was usually provided during the period of lockdown to prevent aerosol-forming treatment and prevent transmission. The tooth was assessed for the prognosis if found to be hopeless and nonrestorable. The patient was referred to the Department of Oral and Maxillofacial Surgery for extraction considering the urgency. The pulpal treatment was offered by either staff members, postgraduate students, or interns of Department of Conservative Dentistry and Endodontics during the entire period of the study.

General considerations and patient triage at the hospital reception was as follows:

  Patient assessment
(1) Patients were asked to wash hands with soap and water for 60 seconds at the main entrance
(2) Patients were asked to rub the hands with alcohol based hand sanitizer for at least 30–40 seconds
(3) Temperature was recorded with a contactless thermometer
(4) Oxygen saturation using pulse oximeter was checked
(5) Status on Aarogya Setu application was checked to describe the patient in terms of low, medium, or high risk of getting infected
(6) Any symptoms for COVID-19 were asked
(7) Strict social distancing by sitting 6 feet apart at the case issuance area was followed
(8) It was compulsory to wear masks and head caps
(9) In the general diagnosis department, a complete verbal history was recorded for each patient including the patient's medical history, travel history, and history of COVID-19-positive in family

A series of questions that were asked as follows:

(1) Are you a suspected/recovered COVID-19 patient?
(2) Have you been in contact with a confirmed/laboratory confirmed COVID-19 patient in the past 2 weeks?
(3) Have you been showing any symptoms of COVID-19?
  - Fever
  - Sore throat
  - Body ache
  - Vomiting
  - Diarrhea
  - Loss of taste or smell
(4) Any travel history in last 15 days?
(5) Are you a healthcare worker on COVID-19 duty?

A subject who provided positive response to any of the above questions was asked to get the COVID-19 test by RAT (rapid antigen test) (SD biosensor test kit, India) before getting examined and then was checked by staff members, interns, or postgraduate students in person. If found positive at this point, they were immediately asked to report to the nearest Urban Health Centre (UHC) for isolation and further treatment if required [9].

Patients entering the department were asked about the history of pain including the origin, duration, type, aggravating and relieving factors, and swelling if any associated, based on which the diagnosis was made and prognosis was assessed following all the departmental protocols as described.

### 2.1. Departmental Protocol

(1) SpO_2_ and temperature was again accessed on entering the department
(2) A mobile screen barrier 4 ft *∗* 4 ft in size was placed while asking the patient about the chief complaint and history who is on the other side of the screen
(3) Two well-ventilated chairs were allotted for doing the diagnosis of the patients
(4) A total of 2 interns, 1 postgraduate student, and 1 professor were allotted for diagnosing with shift-wise replacement to prevent continuous exposure to the staff:
  - The patient was given the needful palliative treatment after complete examination
  - If the patient needed any radiograph, OPG or CBCT was the preferred method to prevent contamination with the oral saliva, and then treatment protocol was decided
  - Pulp sensitivity test was done whenever needed
  - RVG was taken with utmost care to prevent any transmission by using 2-cover technique: finger of nitrile glove covered with receptor sleeve
  - The priority was given more to palliative treatment followed by using hand instruments to prevent aerosol generating procedures
(5) Air conditioners were rarely used
(6) Clear film (Oro Dental Inc., India) was placed on the chair, and the chair was disinfected after each patient
(7) Microscope was covered with a customized OHP sheet just beneath the eyepieces and discarded after every use

### 2.2. Dentist Protocol

1. It is fully equipped with fluid-resistant personal protective equipment (PPE) kit with gown, shoe cover, N95 mask/respirator, head cap, face shield, protective eyewear, and surgical gloves
2. Disinfection of all the surrounding areas with sodium hypochlorite was done after each patient
3. Four-handed dentistry was appreciated with use of PPEs
4. The use of rubber dam was considered mandatory for all the procedures done

### 2.3. Disinfection Protocol: According to IFEA, IES, and IDA Guidelines

1. Sweeping with broom was avoided and use of wet mob was encouraged
2. Toilets, washbasin, and dental chair side basin were regularly cleaned with 1% sodium hypochlorite
3. The healthcare workers were instructed to wear proper gown/PPE with surgical gloves, shoe cover, mask, and cap to prevent infection for cleaning the hospitals
4. Proper time was provided for easy air circulation for at least 15 min after non-AGP and 30 minutes after AGP treatment [10]

### 2.4. Protocol during Lockdown

(1) In order to reduce the risk of infection, the basic screening department was avoided, and the Department of Oral and Maxillofacial Surgery was converted into General Dentistry
(2) Patient is then referred to the specific section as per the complaint
(3) Associated to the Department of Conservative Dentistry, Endodontics, and Aesthetic Dentistry, various protocols are followed:
  - Patients were asked to wear masks until the doctor checks for the intraoral examination
  - All patients had to mandatorily rinse their oral cavities with Betadine gargles for at least 30 secs
  - X-rays and all the AGP treatments were avoided until deemed necessary
  - Palliative treatment protocol was followed to minimize the risk of transmission
  - All AGPs were performed with an additional extraoral Hi-Vac suction system (Eighteeth Medical Inc.)

### 2.5. Protocol for Sequential Unlock

1. When the sequential unlock began, treatment was started in the specific department
2. During the period of Unlocks 1, 2, and 3, all the basic necessary protocols followed were the same, and gradually non-AGP treatments were started along with limited AGP unless there is severe emergency condition with proper precautionary measures taken
3. During the period of Unlocks 4 and 5, the necessary AGP treatments were started with proper precautionary measures taken

### 2.6. General Protocol Followed before Oral Examination of Patients

All the patients were asked to wear the mask until the dentist approaches the patient. Patients were asked to rinse the oral cavity with 0.2% povidone iodine solution for 1 minute. For conducting the oral examination of the patients, the healthcare professionals were made fully equipped with a full PPE kit and all procedures under strict isolation using dental dam.

### 2.7. Stagewise Treatment Protocol

The treatments provided are divided into two types:

(a) Non-AGPs:
  1. Palliative treatment
  2. Temporary filling
  3. Incision and drainage
  4. Pulp capping
(b) AGPs:
  1. Pulpotomy
  2. Pulpectomy

### 2.8. Non-AGPs

#### 2.8.1. Palliative Treatment (PT)

During the period of lockdown and unlock, due to decreased use of AGP treatments, non-AGPs were widely used. Palliative treatment was the most widely provided treatment followed by other non-AGP treatments, and in emergency cases where the palliative treatment failed or patient appeared with severe pain, then AGPs were done.

(a) Analgesics were given as follows:
For mild pain:
(i) Tab Ibuprofen 400 mg (1 tablet taken 4 times a day possibly after food)
OR
(ii) Tab Paracetamol 500 mg (2 tablets taken 4 times a day) (maximum 4 grams a day)
For moderate pain:
(iii) Tab Ibuprofen 400 mg (1 tablet taken 4 times a day preferably after food) (Maximum 2.4 grams) + Tab Paracetamol 500 mg (2 tablets 4 times a day alternatively after 2 hours)
OR
(iv) Tab Diclofenac Sodium 50 mg (1 tablet three times a day (150 mg))
For severe pain:
(v) Tab Ibuprofen 400 mg (1 tablet taken 4 times a day preferably after food) (maximum 2.4 grams) + Tab Paracetamol 500 mg (2 tablets 4 times a day) alternatively
Tab Diclofenac Sodium 50 mg (1 tablet three times a day) (150 mg)
If still not cured referral for emergency treatment.
Precautions:
In case when patients gave history of hypersensitivity or acidity or ulcers in stomach after taking medications, patients were prescribed Antacids or proton pump inhibitors such as Pantoprazole, Rabeprazole, and Omniprazole before lunch followed by taking antibiotics or analgesics [11]
(b) Antibiotics were given as follows:
Indications of antibiotics:
  1. Presence of infection
  2. Systemic involvement of dental infection
  3. Where immediate drainage is not possible
Rx
    Tab Amoxicillin 500 mg (1 tablet taken 3 times a day for 5 days)
    Tab Augmentin 625 mg (1 tablet taken 2 times a day for 5 days)
    Tab Metronidazole 400 mg (1 tablet taken 3 times a day for 5 days) (for anaerobic infection)
    Tab Clindamycin 150 mg (1 tablet taken 4 times a day for 5 days)
    Tab Amoxycillin + Clavulanic Acid, Tab Amoxycillin, Tab Metronidazole acts as first-line antibiotics [11]

#### 2.8.2. Temporary Filling (TF)

In cases, where there was a dull aching and continuous pain or old restorations (broken or with secondary caries) where permanent restoration could not be provided, the carious portion was removed using spoon excavator and restored with interim restoration Cavit (3M, USA). The patient was kept on regular follow-up basis.

#### 2.8.3. Incision and Drainage (ID)

Those patients that appeared with localised intraoral swelling associated with specific tooth; palliative treatment did not prove to be effective; hence, it required pus to be drained out. Nerve block anaesthesia was given and incision on the most dependent part was made, and all the pus was drained out. The patient was prescribed palliative treatment and kept on follow-up.

#### 2.8.4. Pulp Capping (PC)

**Figure 1:**
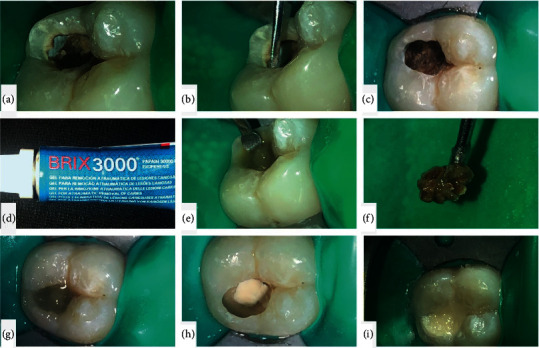
Stepwise procedure of performing pulp capping: Step a, isolation of the affected tooth using dental dam. Step b, removal of the friable margins. Step c, after removal of the friable margin and preparing the cavity for using ART material. Step d, papain gel as a material for performing ART. Step e, application of the papain gel. Step f, excavated carious portion. Step g, reapplication of the papain gel. Step h, pulp capping with MTA base. Step I, restoration of the cavity using capsulated GIC cement.

Using hand instruments like spoon excavator along with papain gel (Brix3000, Brix S.R.L, Argentina), the soft and infected carious was removed. In case of a pinpoint pulp exposure, capping was done using fast setting calcium hydroxide Dycal (Dentsply Sirona, Dentsply Caulk, USA) or mineral trioxide aggregate (Prevest DenPro Ltd., India) which was restored using fluoride releasing cements GC Fuji IX GP (GC Corporation, Tokyo, Japan) as it prevents the caries spread and provide mineralisation of tissue (Figures 1(a)to 1(i)).

### 2.9. AGPs

#### 2.9.1. Pulpotomy (PO)

**Figure 2:**
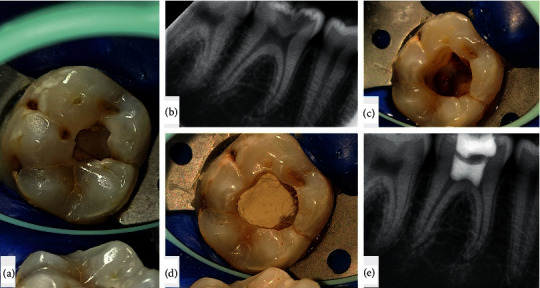
Stepwise procedure of pulpotomy. Step a, preoperative picture of the affected tooth. Step b, preoperative radiograph of the tooth. Step c, removal of the affected coronal pulp and leaving the radicular pulp intact. Step d, placement of MTA. Step e, postoperative radiograph.

In those patients exhibiting severe pain and acute symptoms and their radiographs showing infected coronal pulp, the infected part was removed using a slow speed carbide bur; bleeding was stopped and a Dycal (Dentsply Sirona, Dentsply Caulk, USA) or mineral trioxide aggregate plug (Prevest DenPro Ltd., India) was placed, and interim restoration of Cavit (3M, USA) was done. The patient was kept on follow-up later for replacement with light cure GIC (Glass Ionomer Cement) (GC Corporation, Tokyo, Japan) or composite (Ivoclar Vivadent, Switzerland) (Figures 2(a)to 2(e)).

#### 2.9.2. Pulpectomy (PU)

In case of severe pulpal symptoms along with suspected periapical involvement, a single-sitting pulpectomy was done. The cavity was deroofed using a slow speed carbide bur, and using a single-use NiTi file (Protaper Gold, Dentsply, Maillefer, Switzerland), the entire pulp was extirpated. In case of weeping canals, long-term intracanal calcium hydroxide medicament Metapex (Meta Biomed Inc., Korea) and Cavit (3M, USA) was used as an interim restoration, and the patient was kept on follow-up for later restoration using either light cure GIC (GC Universal Restorative, GC Corporation, Tokyo, Japan), Composite (Ivoclar Vivadent, USA).

## 3. Results

Out of 15052 new patients that reported to our hospital, 5698 appeared to the Department of Endodontics during the entire period of 8 months from March to October 2020 divided into 3 phases mainly prelockdown, lockdown, and sequential unlock. The percentage of endodontic emergencies encountered was 36.10% (971/2690) in prelockdown, 32.65% (368/1127) in lockdown, and with little rise to 38.80% (4359/11235) in the sequential unlock phases representing an overall percentage of 37.85% (5698/15052). Further in the unlock phases, endodontic emergencies were reported to be 656/1707 (38.43%) in Unlock 1, 911/2102 (43.345%) in Unlock 2, 784/1960 (40%) in Unlock 3, 1008/2627 (38.37%) in Unlock 4, and 1000/2839 (35.22%) in Unlock 5.

From the cumulative data that was obtained, it was found out that an overall number of females (*n* = 3264) were more than that of the males (*n* = 2434) (∆ = 830). In the entire duration of 8 months, there was not a single month depicting more number of males. Hence, the results were further compared with Pearson chi-square test, and the difference was found to be statistically insignificant (*P* > 0.05). Similarly, when the male : female ratio was compared internally amongst sequential unlock phases, it was statistically insignificant (*P*=0.83) (Table 1).

A maximum number of patients that approached the department belonged to the age group of 26–35 years during the entire period of prelockdown (278/971), lockdown (130/368), and sequential unlock (1216/4359), and the least approached patients were belonging to the age group of more than 75 years of age constituting (32/971) in prelockdown, (3/368), during lockdown, and (90/4359) during sequential unlock. A high statistically significant difference was observed (*P* < 0.001) through a chi-square test when all the age groups were further compared, and a similar pattern was also observed during various phases of sequential unlock (*P*=0.01), the results of which are shown in Table 2.

**Figure 3:**
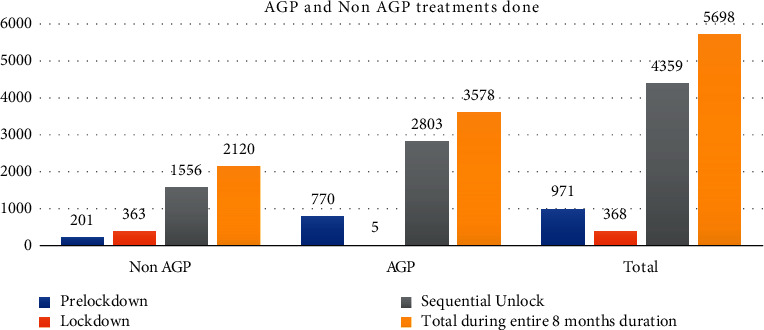
Total number of AGPs and non-AGPs done and comparing them among various time periods and to the total.

The treatments provided were further divided into AGPs and non-AGPs. Non-AGPs were performed where 20.7% during prelockdown were followed by a steep rise of 98.64% during lockdown and a consistent decline to 35.69% during sequential unlock. A graphical representation of the distribution of the number of AGP and non-AGP treatments is as depicted in Figure 3.

A further insight into the individual procedures performed showed that maximum treatment performed was pulpectomy during prelockdown (463/971) as well as sequential unlock (1553/4359), while palliative treatment (231/368) was the maximum performed procedure during lockdown. A highly significant result was observed statistically when the number of treatments provided during the three time periods was compared using chi-square test (*P* < 0.001). Similar findings were observed amongst the unlock phases, wherein palliative measure turned out to be the most performed procedure during Unlock Phase 1 (*n* = 285/656), which later shifted to pulpectomy during the rest of the unlock phases (*n* = 1516/3703) (Table 3).

## 4. Discussion

Dentists are more susceptible to infection because they work in a vulnerable environment, the oral cavity which is considered as the hub of viruses and bacteria. Hence, during this period of COVID-19 pandemic, it has become exceedingly important to rule out the emergency treatment required for the patients and decrease the exposure by focusing more on palliative method of treatment. Our institution is affiliated to Ahmedabad Municipal Corporation and only hospital working round the clock during this pandemic, where more than half of the staff members, interns, and postgraduate students were posted in the field as frontline warriors since 14^th^ March 2020, and the remaining members were working in the institution for providing dental treatment to the patients on a rotational basis to reduce the overall exposure.

A complete shutdown was introduced in India from March 23, 2020. Consequently, all nonemergency services, including dental clinics, were ordained to stop in order to inhibit the transmission of the virus. Our hospital, which is an affiliated tertiary hospital, was open during that period of time. It can be said that the overall patient flow to the hospital has reduced, but due to the shutdown of all the other clinics, the overall percentage of patients remained almost the same thereby increasing our burden for providing the treatment.

A similar hospital-based study was conducted by Sidhartha et al. in India on 247 patients during the period of March 23 to May 31, 2020, focusing more on the diagnostic part of the type of endodontic emergencies encountered and very little on the treatment provided in their study. More number of males appeared to that of females, and the number of patients approaching their hospital increased as the rest of the private clinics were shutdown. Nonsurgical endodontic treatment was the most performed treatment further followed by pulpotomy describing similarity to our study as more of non-AGPs were done during the lockdown and later on gradual shift to AGP with gradual release. [12].

The first wave of COVID-19 hit India in March 2020 and was described as prelockdown, lockdown, and sequential unlock. A hospital-based study was done by Maria et al. in San Paolo Hospital in Milan in Italy where various phases such as prelockdown, lockdown, reopening, and second wave showed similarity of decrease in the number of patients encountered during the period of lockdown and second wave with rebound in reopening focusing more on the type of endodontic emergency encountered and dividing the ages mainly into children, adults, and elderly. Difference was encountered as higher number of males approached compared to that of the females in all the age groups during the lockdown which reversed in elderly during reopening and second wave, whereas our hospital had encountered more of the females irrespective of any age group. [13].

Teledentistry proved to be one of the most effective measures taken during the time of COVID-19 pandemic. J Beauquis et al. conducted a study in Cliniques Universitaires Saint-Luc in Belgium describing the data from April 1, 2020, to April 30, 2020, with a total sample size of total 570 patients with teledentistry done. 49.3% of patients were treated remotely without the need of urgent hospitalisation through teledentistry. [14] Similarly, Jessica et al. conducted a study in Tufts University School of Dental Medicine, USA, from March 30, 2020, to May 8, 2020, where there were a total of 466 patients out of which 199 were treated through teledentistry, and the rest were clinically encountered [8]. It was not undertaken at our hospital as it is the only hospital working and treating patients in person and the other dental healthcare clinics and hospitals were nonfunctional and performing teledentistry. However, departmental teledentistry was functional.

As a result of complete closure, the definition of endodontic emergencies has changed focusing more on irreversible pulpitis, acute periapical abscess, and being a specialist in Endodontics and Conservative Dentistry, the utmost aim of us is salvaging the tooth by undergoing the best treatment possible. Focusing more on the treatment that was provided, palliative treatment was considered to be the primary treatment during the period of lockdown. Several other treatment modalities were used in the event of failure of primary treatment protocol. Rubber dam and extraoral high vacuum evacuators proved to be very useful in reducing transmission. Chemicomechanical means of caries removal, that is, papain gel, was used for carious removal, which was further restored using various fluoride-releasing cements. Hand instruments like spoon excavators were used to remove the soft caries and restored using temporary restoration in order to halt the use of AGPs.

Non-AGPs were the most accepted treatment protocol followed to reduce the transmission, but various AGPs were also used in emergency situations. The microbial burden must be reduced to stop the evaluation of the infection. Emergency pulpotomy was done in severely inflamed pulp, where pulp was amputated and fast setting base of either calcium hydroxide or MTA was provided. Patients with an acute periapical abscess or history of swelling that does not subside on medication with periapical infection need the drainage of the pus to stop the further spread of infection. Emergency pulpectomy was done using a single use rotating NiTi file as its use ensures a good ease of use with a moderate resistance, to allow easy access and initial shaping of the root canal system without further risks and even avoiding the need of sterilization and disinfection, which further proves effective in stopping the chain of transmission [15]. Pulp capping was done in deep carious lesions with pin point pulp exposure, which was sealed by applying base of Dycal forming a calcific barrier and restoring it with fluoride-releasing cements, which helps prevents further spread of the caries.

All the studies conducted so far have found more number of males, but our study depicted the opposite with more number of female patients appeared which is a bit shocking as with the complete shutdown and the females mostly being at home, the patients approaching were expected to be vice versa. The maximum number of patients belongs to the age group of 25–35 years because they were the patients demanding the need to salvage the tooth and concerned with more aesthetics, and the least number of patients was belonging to the age group of more than 75 years as the teeth may show hopeless prognosis describing the need for extraction or surgical intervention. With our hospital located in the heart of the Ahmedabad, a maximum number of patients appearing are having a low socioeconomic status, and most of the patients above the age of 60 years that were not ready to salvage the tooth or not ready with longer visits were then referred to the Department of Oral Surgery for extraction. Jingjing Yu et al. did a study which includes the data of a total 96 patients over a period of 10 days from 22 February, 2020, to 2 April, 2020, in Wuhan University, where the maximum number of patients was in the age group of 45–64 years age group further focusing more on the type of dental emergency and not on the treatment provided. [16].

The second wave of COVID-19 hit India in April 2021, again requiring the use of only emergency treatments and possibly non-AGPs and riding back to increasing the use of PPEs. Patients' flow has declined again, and more than 40% of interns are assigned as frontline warriors. The second wave has hit the India harder compared to the earlier one with approximately 25% of the interns, postgraduate students, and staff members affected by COVID-19 infection, which was very less during the first wave.

There were two major limitations in the study, one being that we only used the data of new patients coming to the department. However, this can be ruled out as our intention was to describe the measures taken and treatments provided during the first visit to the department in such critical times and not during the follow-ups. Another limitation was the duration of this study being written, but since this is a sensitive data of numbers that could only be disseminated after taking numerous internal permissions from the concerned bodies, we did our best to present it at the earliest which may help in the case of future attacks if any.

## 5. Conclusion

During the duration of COVID-19 pandemic, the dentists all around the world not only proved themselves by being true corona warriors but also treated the patients in that space which is most prone to infection and that is the oral cavity. During that time, more than half of our interns, postgraduate students, and staff members were posted on COVID-19 duty, and the remaining interns, postgraduate students, and staff members were working at the hospital premises and serving the patients in the best possible way with not a single day of complete shutdown of our tertiary healthcare hospital. The pandemic had turned the lives of the people upside down with most of the emergencies encountered in dentistry found to be endodontic in origin. The most common patients appeared to belong to the age of 26–35 years, and females were more than males with non-AGPs that were widely provided treatment at the time of lockdown with gradual shift observed towards the AGPs with sequential unlock. Palliative treatment was the primary treatment provided at that time since there was reduction in providing AGPs considering the immense risk of contracting the virus, and it was hence provided as and when needed in emergency.

Although the results of this retrospective study are of the first wave of COVID-19, the study would describe the immediate measures taken by a tertiary dental care hospital in Ahmedabad, India, adopting a new normal and then continuing it and utilizing the protocols as a lesson learned during the second wave that hit even harder and proving to be a guidebook preparing us against any unforeseen calamities.

## Figures and Tables

**Table 1:** Genderwise distribution of patients during various time periods.

Time periods	Male *n* (%)	Female *n* (%)	Total *n* (%)	*χ* ^2^ (*P* value)
Prelockdown	384 (39.55)	587 (60.45)	971 (17.04)	4.84 (0.09)
Lockdown	158 (42.93)	210 (57.07)	368 (6.46)	
Sequential unlock	1892 (43.40)	2467 (56.60)	4359 (76.50)	
Total *n* (%)	2434 (42.72)	3264 (57.28)	5698 (100.0)	

Gender-wise distribution of patients during sequential unlock				
Unlock 1	278 (42.38)	378 (57.62)	656 (15.05)	1.47 (0.83)
Unlock 2	407 (44.68)	504 (55.32)	911 (20.90)	
Unlock 3	336 (42.86)	448 (57.14)	784 (17.99)	
Unlock 4	445 (44.15)	563 (55.85)	1008 (23.12)	
Unlock 5	426 (42.60)	574 (57.40)	1000 (22.94)	
Total *n* (%)	1892 (43.40)	2467 (56.60)	4359 (100.0)	

**Table 2:** Agewise distribution of patients during various time periods.

Time periods	15–25 *n* (%)	26–35 *n* (%)	36–45 *n* (%)	46–55 *n* (%)	56–65 *n* (%)	>75 *n* (%)	Total *n* (%)	*χ* ^2^ (*P* value)
Prelockdown	202 (20.80)	278 (28.63)	227 (23.38)	135 (13.90)	97 (9.90)	32 (3.30)	971 (17.04)	38.63 (<0.001)^*∗∗*^
Lockdown	78 (21.19)	130 (35.33)	83 (22.55)	51 (13.86)	23 (6.25)	3 (0.82)	368 (6.46)	
Sequential unlock	1055 (24.20)	1216 (27.90)	1050 (24.09)	675 (15.48)	273 (6.26)	90 (2.07)	4359 (76.50)	
Total *n* (%)	1335 (23.43)	1624 (28.50)	1360 (23.87)	861 (15.11)	393 (6.90)	125 (2.19)	5698 (100.0)	

Sequential unlock	15–25 *n* (%)	26–35 *n* (%)	36–45 *n* (%)	46–55 *n* (%)	56–65 *n* (%)	>75 *n* (%)	Total *n* (%)	*χ* ^2^ (*P* value)

Agewise distribution of patients during sequential unlock								
Unlock 1	156 (23.78)	205 (31.25)	166 (25.31)	84 (12.80)	32 (4.88)	13 (1.98)	656 (15.05)	38.37 (0.01)^*∗*^
Unlock 2	196 (21.51)	260 (28.54)	236 (25.91)	137 (15.04)	58 (6.37)	24 (2.63)	911 (20.90)	
Unlock 3	193 (24.62)	221 (28.18)	196 (25)	124 (15.82)	45 (5.74)	5 (0.64)	784 (17.99)	
Unlock 4	237 (23.51)	280 (27.78)	220 (21.83)	173 (17.15)	69 (6.85)	29 (2.88)	1008 (23.12)	
Unlock 5	273 (27.3)	250 (25)	232 (23.2)	157 (15.7)	69 (6.9)	19 (1.9)	1000 (22.94)	
Total *n* (%)	1055 (24.20)	1216 (27.90)	1050 (24.09	675 (15.49)	273 (6.26)	90 (2.06)	4359 (100.0)	

**Table 3:** Treatmentwise distribution of patients during various time periods.

Time periods		PT *n* (%)	PO *n* (%)	PU *n* (%)	TF *n* (%)	ID *n* (%)	PC *n* (%)	Total *n* (%)	Χ^2^ (*P* value)
Prelockdown		34 (3.51)	307 (31.62)	463 (47.68)	81 (8.34)	22 (2.26)	64 (6.59)	971 (17.04)	1040 (<0.001)^*∗∗*^
Lockdown		231 (62.77)	3 (0.82)	2 (0.54)	16 (4.35)	20 (5.43)	96 (26.09)	368 (6.46)	
Sequential unlock		593 (69.11)	1250 (80.13)	1553 (76.96)	403 (80.60)	122 (74.39)	438 (73.24)	4359 (76.50)	
Total *n* (%)		858 (15.06)	1560 (27.38)	2018 (35.42)	500 (8.78)	164 (2.88)	598 (10.49)	5698 (100.0)	

Sequential unlock	PT *n* (%)		PO *n* (%)	PU *n* (%)	TF *n* (%)	ID *n* (%)	PC *n* (%)	Total *n* (%)	Χ^2^ (*P* value)

Treatmentwise distribution of patients during time sequential unlock									
Unlock 1	285 (34)		34 (5.18)	37 (5.64)	53 (8.08)	25 (3.81)	222 (33.84)	656 (15.05)	1368 (<0.001)^*∗∗*^
Unlock 2	50 (5.49)		318 (34.91)	395 (43.36)	83 (9.11)	33 (3.62)	32 (3.51)	911 (20.90)	
Unlock 3	25 (3.19)		276 (35.20)	344 (43.88)	72 (9.18)	28 (3.27)	39 (4.97)	784 (17.99)	
Unlock 4	141 (13.99)		308 (30.55)	372 (36.90)	94 (9.32)	16 (1.59)	77 (7.64)	1008 (23.12)	
Unlock 5	92 (9.20)		314 (31.40)	405 (40.50)	101 (10.1)	20 (2.00)	68 (6.80)	1000 (22.94)	
Total *n* (%)	593 (13.60)		1250 (28.68)	1553 (35.63)	403 (9.25)	122 (2.80)	438 (10.05)	4359 (100.0)	

## Data Availability

All data generated or analysed during this study are included in this article, and additional datasets generated during the current study are available from the corresponding author on reasonable request.

## Additional Points

*Significance for Public Health.* COVID-19 pandemic significantly affected professionals of all fraternities worldwide. Since SARS-CoV-2 is highly infective through airborne contamination, the high risk in dental environment is of serious concern for both professionals as well as patients. Dental services in the time of first wave of COVID-19 proved to be a challenge as most of the setups had shut themselves down considering the novelty of the virus. Delivering services in such hard times was no less than a challenge, that too following all the precautions in set-up where half of the manpower was constantly posted in duties citywide on field as well as inwards and hospitals. This study would stand out as a guidebook for further waves if any yet to come, and also would ease out the preparedness.
